# High-Calorie Diet Consumption Induces Lac-Phe Changes in the Brain in a Time-of-Day Manner Independent of Exercise

**DOI:** 10.3390/metabo15060375

**Published:** 2025-06-06

**Authors:** Jarne Jermei, Han Jiao, Ayano Shiba, Julia C. Goedhart, Roberta Tandari, Andries Kalsbeek, Eduard A. Struys, Chun-Xia Yi

**Affiliations:** 1 Department of Endocrinology and Metabolism, Amsterdam University Medical Center, Location AMC, University of Amsterdam, 1105 AZ Amsterdam, The Netherlands; j.jermei@amsterdamumc.nl (J.J.); h.jiao@amsterdamumc.nl (H.J.); a.shiba@nin.knaw.nl (A.S.); j.goedhart@nin.knaw.nl (J.C.G.); r.tandari@amsterdamumc.nl (R.T.); a.kalsbeek@amsterdamumc.nl (A.K.); 2 Amsterdam Gastroenterology, Endocrinology and Metabolism, 1105 AZ Amsterdam, The Netherlands; 3 Department of Clinical Chemistry, Laboratory of Endocrinology, Amsterdam University Medical Center, Location AMC, 1105 AZ Amsterdam, The Netherlands; 4 Netherlands Institute for Neuroscience, 1105 BA Amsterdam, The Netherlands; 5 Department of Laboratory Medicine, Amsterdam University Medical Center, Location AMC, 1105 AZ Amsterdam, The Netherlands; e.struys@amsterdamumc.nl

**Keywords:** Lac-Phe, *CNDP2*, *SLC16A1*, brain, microglia, exercise, time-restricted feeding, high-calorie diet, circadian rhythm

## Abstract

**Background/Objectives:** N-lactoyl-phenylalanine (Lac-Phe), an exercise-induced metabolite, has been shown to reduce food intake, decrease body weight and adiposity, and improve glucose homeostasis without affecting energy expenditure. Until now, Lac-Phe has mainly been investigated in blood plasma, showing its appetite-suppressing effects. Interestingly, these beneficial effects were caused by a temporary increase in Lac-Phe levels after exercise. Second, despite the central role of the central nervous system in the homeostatic control of energy metabolism, little is known about the presence and function of Lac-Phe in the brain. The goal of this study is to investigate how Lac-Phe concentrations in the brain change during the 24 h light/dark cycle. **Methods**: We conducted an experiment in rats in which time-restricted running was combined with time-restricted feeding (TRF) of a high-calorie diet, after which Lac-Phe levels were measured in the hypothalamus and cortex using stable isotope dilution LC-MS/MS. Microglia were isolated from rat brains to study Lac-Phe-related gene expression. **Results**: We found that Lac-Phe levels changed over time within the 24 h light/dark cycle in the hypothalamus and/or cortex, even without exercise. Our study indicates that brain Lac-Phe is not only induced by exercise but also by high-calorie diet intake independent of exercise. Finally, we showed that microglial cells are cytosolic nonspecific dipeptidase 2 (*CNDP2*) positive and therefore able to produce Lac-Phe. Hereby, we identified *SLC16A1* in microglia as a possible key mediator of Lac-Phe production. **Conclusions**: We conclude that high-calorie diet consumption induces Lac-Phe changes in the brain in a time-of-day manner independent of exercise. This study provides new knowledge on the presence and production of Lac-Phe in the brain. Further research is needed to elucidate the potential mechanism by which Lac-Phe reduces food intake and body weight by targeting appetite-suppressing neurons.

## 1. Introduction

Globally, the prevalence of obesity has considerably increased in the past decades and will only continue to rise if no major changes are made [1,2,3]. Obesity is a major health problem since it elevates the risk of developing metabolic diseases such as type 2 diabetes, which is a leading cause of death worldwide [2,4]. In addition to reducing caloric intake, the second most important recommendation for managing obesity and its associated metabolic diseases is physical exercise [5,6]. Specifically, exercise promotes body weight reduction and loss of body fat, as well as improved insulin sensitivity [6,7].

Recently, N-lactoyl-phenylalanine (Lac-Phe) has been identified in the blood as a new exercise-induced metabolite that could play a significant role in the protective effects against obesity [8,9]. Upon exercise, Lac-Phe is synthesized by cytosolic nonspecific dipeptidase 2 (CNDP2)-positive cells across the body including the brain, such as macrophages and epithelial cells, from L-Lactate, known to be increased after exercise, and L-Phenylalanine [8,10]. Lac-Phe has been shown to reduce food intake, decrease body weight and body adiposity, and improve glucose homeostasis, without affecting energy expenditure [8,9].

Until now, Lac-Phe has mainly been investigated in blood plasma, showing its appetite-suppressing effects [8]. Interestingly, these beneficial effects seem to be caused by a temporary increase in Lac-Phe levels after exercise [8]. Second, it remains unclear how Lac-Phe regulates appetite and which brain mechanisms are involved. Potential mechanisms that have been previously discussed include targeting appetite-regulating neurons with Lac-Phe through possible endocrine and/or paracrine functions [11]. Few studies have examined the possible presence of Lac-Phe in the brain [8,10]. Furthermore, Lac-Phe concentrations in the hypothalamus of exercised mice were compared to those of sedentary mice without any differences, but further evidence on the presence of Lac-Phe in the brain is lacking [8]. 

Because of the appetite-suppressing effects caused only by temporary increases in blood Lac-Phe, in addition to a lack of knowledge on the presence and function of Lac-Phe in the brain, the goal of this study was to investigate how Lac-Phe concentrations change during the 24 h light/dark (L/D) cycle and to gain more insight into the duration of Lac-Phe elevation in the brain. 

## 2. Materials and Methods

### 2.1. Study Design

To investigate our research question, we conducted two experiments in rats. In the first experiment, rats were subjected to time-restricted running combined with time-restricted feeding (TRF) of a high-calorie diet. Lac-Phe levels in brain tissue were measured using stable isotope dilution LC-MS/MS. In the second experiment, rats received TRF of a high-calorie diet, and microglia were isolated to analyze Lac-Phe-related gene expression.

### 2.2. Animal Experiments

#### 2.2.1. Experiment-1: Animals for Brain Lac-Phe Measurements

A total of 80 male Wistar rats (Charles River Laboratories, Sulzfeld, Germany) were used for brain Lac-Phe measurements, after conducting an experiment in which time-restricted running was combined with TRF of a high-calorie diet as described earlier [12].

Upon arrival, animals (around 9 weeks old) were group-housed on a 12 h light/12 h dark cycle (lights on at 7:00 a.m.; Zeitgeber time zero [ZT 0]) at a room temperature of 22 ± 2 °C. After 1 week of acclimatization, all animals received ad libitum feeding of a diet containing 59 kJ% fat and 25 kJ% carbohydrates (HFD; E15772-347 EF D12331 mod., ssniff Spezialdiäten GmbH, Soest, Germany) with normal tap water.

After 1 week of acclimatization to the HFD, rats were randomized and pair-housed in custom-made cages [522 (w) × 582 (l) × 412 (h) mm] with a vertical 36 cm diameter stainless-steel running wheel (Model 80850MS, Campden Instruments, Loughborough, UK) or in Conventional EU Type 4 cages without a running wheel. Thereby, animals had either ad libitum access to a running wheel (ALR) or no access to a running wheel (NR).

**Figure 1:**
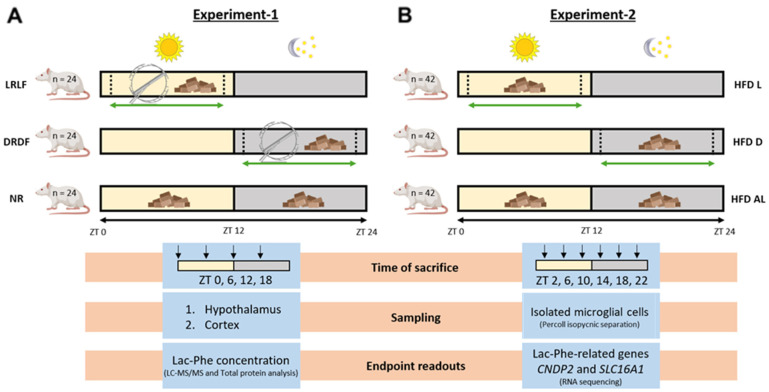
Experimental design. (**A**) Overview of Experiment-1 and (**B**) overview of Experiment-2, including experimental groups and group number, time of sacrifice, sampling, and endpoint readouts with the methods used. LRLF, access to the running wheel restricted to the light period in combination with only light-phase feeding; DRDF, access to the running wheel restricted to the dark period in combination with only dark-phase feeding; NR, no access to a running wheel; HFD L, light-phase feeding of a high-fat diet; HFD D, dark-phase feeding of a high-fat diet; HFD AL, *ad libitum* feeding of a high-fat diet.

After a basal running period of 18 days, ALR rats were further divided into the following experimental groups: access to the running wheel restricted to the dark period in combination with only dark-phase feeding (ZT 13–23; DRDF), and access to the running wheel restricted to the light period in combination with only light-phase feeding (ZT 1–11; LRLF) (Figure 1A). This was achieved by blocking the running wheel using an Arduino-controlled actuator. During the braking procedure, the actuator gradually increased the friction on the running wheel over 20 s until a full brake was accomplished so that the rats would not be injured while running during the braking procedure.

After 4 weeks of time-restricted running and feeding, animals were sacrificed at four different time points along the 24 h L/D cycle (starting at ZT 0, every six hours) by euthanasia with 60% CO_2_/40% O_2_, followed by decapitation. The brain was isolated, dissected, and from one half of the brain, the hypothalamus and part of the cortex were extracted, and immediately snap frozen in liquid nitrogen and stored at −80 °C for further analyses.

#### 2.2.2. Experiment-2: Time-Restricted Feeding Animals

Male Wistar rats (n = 141) (Charles River Laboratories, Sulzfeld, Germany) were group-housed under a 12 h light / 12 h dark cycle at a room temperature of 22 ± 2 °C. Upon arrival, animals (around 7 weeks old) received a HFD (E15772-347 EF D12331 mod., ssniff Spezialdiäten GmbH, Soest, Germany). During an acclimatization period of 4 weeks, the animals had *ad libitum* access to HFD and tap water. Animals arrived and were group-housed in different batches for logistical reasons.

After acclimatization, animals were randomized for TRF, receiving either light-phase feeding (HFD L; 10 h of feeding) under normal L/D conditions (lights on at 7:00 a.m.; ZT 0), dark-phase feeding (HFD D; 10 h of feeding) under reversed L/D conditions (lights on at 7:00 p.m.; ZT 0), or *ad libitum* feeding (HFD AL) under either normal or reversed L/D conditions (Figure 1B). 

After 4 weeks of TRF, animals were sacrificed at six different time points during the 24 h L/D cycle (starting at ZT 2, every 4 h) by euthanasia with 60% CO_2_/40% O_2_, followed by decapitation. The brain was isolated and dissected as previously described [13]. From one half of the brain, the hypothalamus and hippocampus were removed, and the remainder of this half of the brain, together with the cerebral cortex of the other half of the brain, was used for microglia isolation.

### 2.3. Quantitative Lac-Phe Measurement by LC-MS/MS in Hypothalamus and Cortex

Lac-Phe levels in the hypothalamus and cortex were determined using stable isotope dilution LC-MS/MS. After the addition of 50 μL 0.1 μM N-lactoyl-phenylalanine-d5 (Lac-Phe-d5; HB7279, Hello Bio, Dunshaughlin, Ireland) and 200 μL of water containing 0.9% sodium chloride, tissues were homogenized in Fisherbrand Pre-Filled Bead Mill Tubes (Thermo Fisher Scientific, Waltham, MA, USA). After homogenization, 25 μL of the homogenate was used for total protein quantification and the remaining homogenate was used for LC-MS/MS.

Protein quantification was performed using the DC Protein Assay (Bio-Rad; 500-0116, Bio-Rad DC Protein Assay Reagents Package, Hercules, CA, USA) following the manufacturer’s guidelines with different bovine serum albumin concentrations in water, containing 0.9% sodium chloride, as protein standards. Absorbance was measured at 750 nm using a Varioskan Flash Spectral Scanning Multimode Reader (version 40053; Thermo Fisher Scientific, Waltham, MA, USA).

For Lac-Phe measurement by LC-MS/MS, the homogenate was filtered through a 10 kDa centrifugal filter (Amicon Ultra 0.5 mL 10 kDa Centrifugal Filter Regenerated Cellulose 10,000 MWCO, Millipore, Darmstadt, Germany) to remove proteins (20 min, 14,000 rpm, 4 °C). Subsequently, Lac-Phe was measured using a UPLC-MS/MS system consisting of a Vanquish-UPLC system (Thermo Scientific) coupled with a TSQ Quantiva tandem mass spectrometer (Thermo Scientific).

Chromatographic separation was achieved using a Waters BEH-C18 UPLC column (Acquity, Milford, MA, USA) set to 25 °C. Hereby, 23.8 mmol/L ammonium formate (pH 9.5 adjusted with 25% ammonia) was used as mobile phase-A and 100% methanol was used as mobile phase-B. The total run time was 14 min at a flow rate of 0.175 mL/min. A linear gradient was applied as follows: 0 min: 5% mobile phase-B, 1 to 10 min: linear gradient to 50% mobile phase-B, 10.1 to 14 min: 5% mobile phase-B.

**Figure 2:**
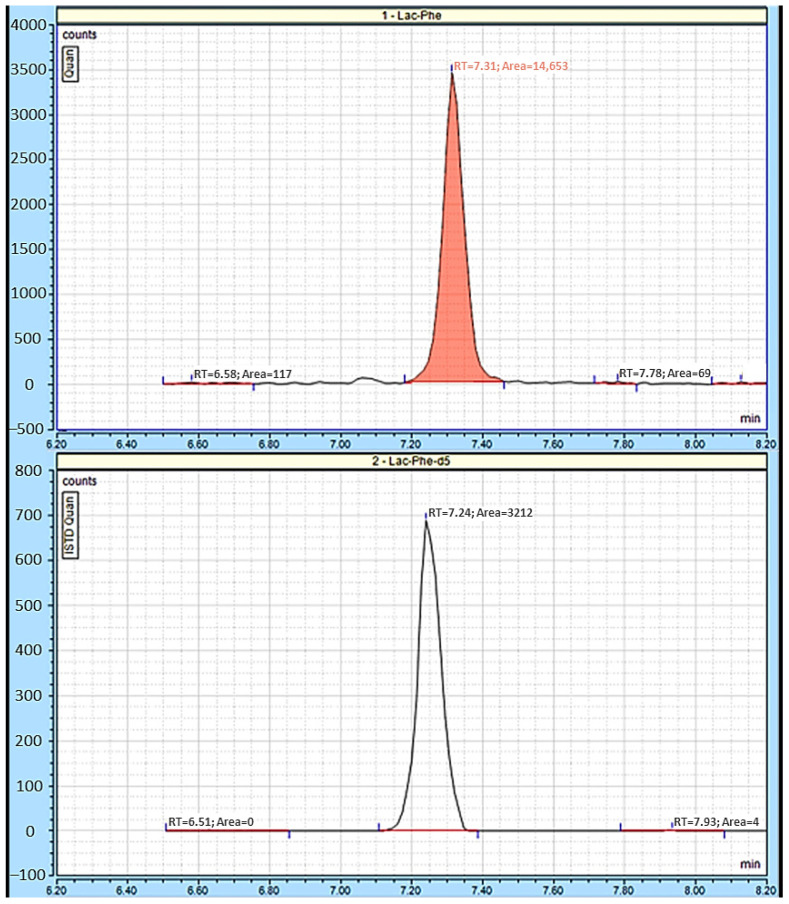
Mass chromatograms of Lac-Phe. (**Top panel**) Chromatogram of endogenous Lac-Phe in a cortical sample. (**Bottom panel**) Chromatogram of the Lac-Phe-d5 internal standard measured in the same cortical sample. Lac-Phe was determined using stable isotope dilution LC-MS/MS. Endogenous Lac-Phe was quantified by comparing the generated area response (**top panel**) using the corresponding stable isotope Lac-Phe-d5 (**bottom panel**) as an internal standard.

Lac-Phe was quantified by comparing the generated area response using the corresponding stable isotope as an internal standard (Figure 2). Finally, the absolute Lac-Phe concentrations from LC-MS/MS analysis were normalized using total protein concentrations to obtain relative Lac-Phe concentrations.

### 2.4. Microglia Isolation

Microglial cells were isolated from one half of the brain, excluding the hippocampus and hypothalamus, and the cerebral cortex of the other half of the brain, using Percoll isopycnic separation, which has been shown to be an efficient method for microglial isolation [14]. Briefly, brains were mechanically homogenized in RPMI 1640 medium (Ref.: 11875-093, Gibco™, Grand Island, NY, USA) using a tissue grinder (Sigma-Aldrich, Toluca, Mexico), after which the brain homogenate was filtered through a 70 μm cell strainer (Ref.: 431751, Corning^®^, Corning, NY, USA) in a 50 mL Falcon tube. The filtered brain homogenate was centrifuged (ROTINA 420 R, Hettich, Germany) for 5 min (380× *g*, 4 °C, brake 9/9). The supernatant was discarded and the pellet was resuspended in 7 mL RPMI medium and mixed with 100% Percoll solution [for 10 mL: 9 mL Percoll^®^ stock (Ref.: 17-5445-01, GE Healthcare, Sigma-Aldrich^®^) together with 1 mL 10× HBSS (Ref.: 14185-045, Gibco™)]. The cell suspension was slowly layered on 70% Percoll solution [for 10 mL: 7 mL 100% Percoll solution with 3 mL 1× HBSS (Ref.: 14175-053, Gibco™)] in a 15 mL Falcon tube and centrifuged for 30 min (500× *g*, 18 °C, brake 1/0). Myelin and cell debris were discarded and the fuse interphase, containing microglial cells, was carefully collected in 8 mL 1× HBSS, followed by centrifuging for 7 min (500× *g*, 18 °C, brake 9/9). The supernatant was discarded and the microglial cell pellet was resuspended in RNA Lysis buffer (Qiagen, Venlo, The Netherlands) and stored at −80 °C until RNA extraction.

### 2.5. RNA Sequencing

RNA from microglial cells was isolated using the RNeasy Micro Kit (Qiagen), according to the manufacturer’s guidelines. mRNA was enriched from total RNA with the Dynabeads^®^ mRNA Purification Kit (61006, Invitrogen, Carlsbad, CA, USA). A strand-specific transcriptome library was constructed using the MGIEasy RNA Directional Library Prep Set, Shenzhen, China (16 RXN) (1000006385, MGI). Briefly, following cDNA synthesis, adaptors were ligated with cDNA fragments. Subsequently, a Polymerase Chain Reaction (PCR) was conducted to amplify the cDNAs, after which the library quality was checked using an Agilent 2100 Bioanalyzer (G2939AA, Agilent, Santa Clara, CA, USA). Finally, single-stranded cyclized products were produced from single-stranded PCR products. Single-stranded circular DNA molecules were replicated via rolling cycle amplification and a DNA nanoball (DNB) was generated. Sequencing was performed using combinatorial Probe-Anchor Synthesis (cPAS) on DNBSEQ-G400 (G400, MGI).

### 2.6. Statistical Analyses

Data are expressed as mean ± s.e.m. Statistical analyses were performed using GraphPad Prism (version 10.2.0, GraphPad Software Inc., La Jolla, CA, USA). Two-way ANOVA was used to assess the effects of *Exercise/Diet*, *Time* (ZT), and their *Interaction*. For Lac-Phe concentrations, a one-way ANOVA followed by Tukey’s multiple comparisons test was applied to assess the effect of *Exercise/Diet* within each time point separately, and to assess the effect of *Time* within each experimental group separately, whereas an unpaired *t*-test was used to evaluate day/night differences. Tukey’s multiple comparison test was used to compare the effects of different dietary conditions at each time point on gene expression levels and the effect of *Time* on gene expression levels within each experimental group. Statistical significance was set at *p* < 0.05.

Sequencing data were filtered using SOAPnuke (v1.5.6) [15] by (1) removing reads containing sequencing adapters; (2) removing reads whose low-quality base ratio (base quality less than or equal to 15) was more than 20%; (3) removing reads whose unknown base (‘N’ base) ratio was more than 5%, after which clean reads were obtained and stored in FASTQ format. Clean sequence reads were aligned to the reference genome (Rattus_norvegicus, GCF_000001895.5_Rnor_6.0) using HISAT2 (v2.1.0) [16], after which Ericscript (v0.5.5) [17] and rMATS (v3.2.5) [18] were used to detect fusion genes and differential splicing genes (DSGs), respectively. Clean sequence reads were aligned to a reference gene set, a database built by BGI (Beijing Genomic Institute in Shenzhen), in which known and novel, coding and non-coding transcripts were included, using Bowtie2 (v2.3.4.3) [19]. The original gene symbols were provided by BGI, after which genes were reannotated using biomaRt, using Ensembl (v109) [20]. The obtained counts of Lac-Phe-related genes *CNDP2* and *SLC16A1* were analyzed using R (v4.1.0, R Foundation, Vienna, Austria). The counts were normalized using the weighted trimmed mean of M-values to the reference with edgeR (v3.6.7) [21] and transformed to log_2_-counts per million [log_2_(CPM)] using voom to estimate the mean–variance relationship.

## 3. Results

### 3.1. Experiment-1: Lac-Phe Levels Change in a Time-of-Day Dependent Manner in the Hypothalamus and Cortex Dependent on Exercise/Diet

**Figure 3:**
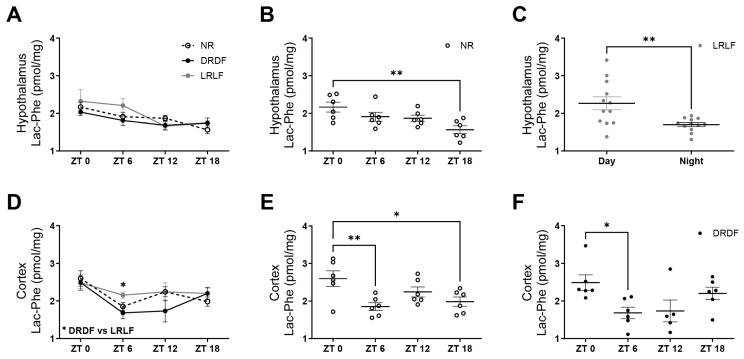
Effect of NR, DRDF, and LRLF on Lac-Phe concentrations in the hypothalamus and cortex over time within the 24 h L/D cycle. (**A**) Effect of no access to a running wheel (NR), access to the running wheel restricted to the dark period in combination with only dark-phase feeding (DRDF), and access to the running wheel restricted to the light period in combination with only light-phase feeding (LRLF) on hypothalamic Lac-Phe over time within the 24 h L/D cycle. (**B**) Effect of *Time* on hypothalamic Lac-Phe in NR rats within the 24 h L/D cycle. (**C**) Day/night differences in hypothalamic Lac-Phe levels in LRLF rats. (**D**) Effect of NR, DRDF, and LRLF on cortical Lac-Phe over time within the 24 h L/D cycle. (**E**) Effect of *Time* on cortical Lac-Phe in NR rats within the 24 h L/D cycle. (**F**) Effect of *Time* on cortical Lac-Phe in DRDF rats within the 24 h L/D cycle. Data are expressed as mean ± s.e.m. Statistical significance was determined using two-way ANOVA to assess the effects of *Interaction, Exercise/Diet, and Time*; one-way ANOVA followed by Tukey’s multiple comparisons test was used to assess the effect of *Exercise/Diet* within each time point separately, and to assess the effect of *Time* within each experimental group separately, whereas unpaired *t*-test was used to evaluate day/night differences (* *p* < 0.05; ** *p* < 0.01).

To evaluate daily changes in brain Lac-Phe concentration, whether or not upon different timed exercise with corresponding timed feeding, Lac-Phe was measured at different time points in the hypothalamus and cortex, brain areas respectively involved in appetite regulation and in the control of locomotor activity (Figure 3). In the hypothalamus, the two-way ANOVA revealed a significant *Time* effect (*p* < 0.001), but no significant *Exercise/Diet* (*p* = 0.274) or *Interaction* (*p* = 0.469) effects (Figure 3A). Regarding the effect of time, Lac-Phe levels in the hypothalamus of NR rats were higher at ZT 0 than at ZT 18 (Figure 3B). Furthermore, in LRLF rats, despite no significant changes between ZT 0, 6, 12, and 18, Lac-Phe showed a significant day/night difference in the hypothalamus (Figure 3C). In the cortex, a significant *Time* effect (*p* < 0.001) was observed, but no significant *Exercise/Diet* (*p* = 0.143) or *Interaction* (*p* = 0.319) effects were found (Figure 3D). However, concerning the effect of exercise/diet at each time point separately, Lac-Phe levels were higher in LRLF rats than in DRDF rats at ZT 6. In addition, similar to Lac-Phe concentrations in the hypothalamus, Lac-Phe was higher in the cortex of NR rats at ZT 0 than at ZT 18, but was also higher at ZT 0 than at ZT 6 (Figure 3E). Concerning the effect of time in the cortex, DRDF rats also showed a higher Lac-Phe concentration at ZT 0 than at ZT 6 (Figure 3F). In general, these results indicate that Lac-Phe levels change over time within the 24 h L/D cycle in both the hypothalamus and cortex, and that these changes may be affected by the timing of exercise/diet.

### 3.2. Experiment-2: Circadian Expression of CNDP2 and SLC16A1 in Microglia After TRF HFD

**Figure 4:**
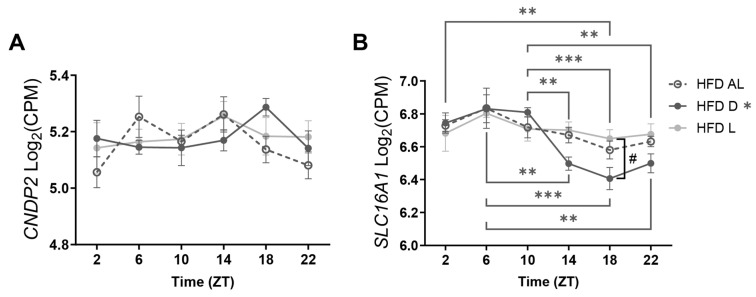
Effect of HFD TRF on Lac-Phe-related genes in microglia over time within the 24 h L/D cycle. (**A**) Effect of HFD ad libitum feeding (HFD AL), HFD dark-phase feeding (HFD D) and HFD light-phase feeding (HFD L) on *CNDP2* gene expression in microglia over time within the 24 h L/D cycle. (**B**) Effect of HFD AL, HFD D, and HFD L on *SLC16A1* gene expression in microglia over time within the 24 h L/D cycle. Data (n = 33–34 per group) are expressed as mean ± s.e.m. (n = 5–6 per group per time point). Statistical significance was determined using two-way ANOVA to assess the effects of *Interaction, Exercise/Diet, and Time*; Tukey’s multiple comparison test was used to compare the effect of the different feeding time conditions at each time point on gene expression levels (^#^ *p* < 0.05) and the effect of *Time* on gene expression levels within each experimental group separately (* *p* < 0.05; ** *p* < 0.01; *** *p* < 0.001). The asterisk next to HFD D indicates a significant effect of *Time* in that group.

Interestingly, in Experiment-1, differences in brain Lac-Phe concentrations were noticed in NR rats (Figure 3B,E), as a result of which we hypothesized that food intake could have an effect on Lac-Phe concentration, independent of exercise. Therefore, the rats in Experiment-2 did not have access to running wheels. We investigated circadian expression of the Lac-Phe-related genes *CNDP2* and *SLC16A1* in microglia from the whole brain of rats on TRF HFD (Figure 4). *CNDP2* is an enzyme responsible for Lac-Phe production. Previous research has shown that, in the brain of rodents, *CNDP2* is higher expressed in microglia than in other cell types [22]. *SLC16A1* has also been shown to be present in microglia and encodes Monocarboxylate Transporter 1 (MCT1), which plays a role in lactate shuttling by transporting lactate into or outside the cell [23,24,25]. In all three experimental groups, *CNDP2* expression remained constant with no significant time-of-day differences (Figure 4A). However, *SLC16A1* expression changed over time within the 24 h L/D cycle in microglia of HFD D animals, whereas no time-of-day changes were found in the microglia of other TRF HFD animals (Figure 4B). Furthermore, microglial *SLC16A1* expression was higher after HFD L than after HFD D at ZT 18. These results confirm that *CNDP2* is expressed in microglial cells and suggest that microglial lactate shuttling fluctuates during the 24 h L/D cycle.

## 4. Discussion

To the best of our knowledge, this is the first report showing that Lac-Phe concentrations in the brain change over time during a 24 h L/D cycle. In addition, we suggest that consumption of a high-calorie diet regulates brain Lac-Phe production independently of exercise, potentially mediated by microglia via the *SLC16A1* lactate transporter gene.

This study investigated changes in Lac-Phe and Lac-Phe-related genes in the brain with a special focus on the effects of diet, exercise and time-of-day. In the first experiment, when time-restricted running was combined with a TRF HFD, we found daily changes in brain Lac-Phe concentrations that seemed independent of exercise. In the second experiment, with only TRF HFD and no exercise, Lac-Phe-related gene expression showed time-of-day dependent changes.

The major strength of this study is the method used to measure Lac-Phe. Here, Lac-Phe was measured in the brain by stable isotope dilution LC-MS/MS, whereas other studies either used untargeted metabolomics to identify Lac-Phe or targeted metabolomics on Lac-Phe without stable isotope internal standards of Lac-Phe, but using standard curves with known Lac-Phe concentrations [8,26,27]. Using our method, we objectively quantified Lac-Phe concentrations for the first time, using the same known internal standard of Lac-Phe.

First, we showed that Lac-Phe concentrations in the brain changed over time during the 24 h L/D cycle. Lac-Phe levels differed in a time-of-day manner in the hypothalamus and/or cortex, independent of the timing of exercise in combination with restricted feeding patterns. Our results also suggest that changes in Lac-Phe concentration might not be induced only by exercise, as initially suggested by previous research [8]. Specifically, we report that non-exercised (i.e., sedentary) rats on HFD AL show time-of-day differences in Lac-Phe concentrations in both the hypothalamus and cortex, although the most pronounced changes were found in the cortex of animals that could only run and eat during the dark period. Therefore, we hypothesized that brain Lac-Phe levels would also be affected by food intake. This idea is supported by a study by *Scott et al.*, who reported higher serum Lac-Phe levels in non-fasted human individuals than in fasted individuals. They speculated that Lac-Phe after eating regulates appetite by acting as a feedback mechanism [26]. Our results are in line with this hypothesis, as we observed higher Lac-Phe concentrations during the day in the hypothalamus of LRLF rats that could eat only during the light period. Cortical Lac-Phe levels were also highest in the middle and end of the night in DRDF rats, whereas in the middle of the light period (i.e., ZT 6) Lac-Phe levels in the cortex were higher in the LRLF rats than in the DRDF rats.

Second, we showed that *CNDP2* was noticeably expressed in microglial cells, independent of the time of day or HFD. This result also confirmed that microglia can produce Lac-Phe [22]. This observation supports the hypothesis that microglia synthesize Lac-Phe and mediate paracrine actions in appetite-regulating neurons [11]. However, from these results, we could not draw any conclusions regarding the amount of Lac-Phe produced. Based on the findings of *Li et al*., Lac-Phe concentrations were not correlated with CNDP2 protein levels, as CNDP2 levels did not change after exercise despite Lac-Phe changes [8].

Nevertheless, plasma Lac-Phe is found to be regulated by extracellular muscle-derived lactate after exercise, suggesting that plasma Lac-Phe levels correlate with extracellular lactate levels after exercise [8,10]. In addition, recent evidence of the effect of metformin, an anti-diabetic drug that reduces body weight, on Lac-Phe has also emerged, concluding that metformin induces Lac-Phe production through intracellular glycolysis-derived lactate in CNDP2+ cells [27]. Considering that Lac-Phe can be produced from both extracellular lactate from muscles and intracellular lactate from glycolysis in CNDP2+ cells, we investigated the 24 h expression of *SLC16A1,* known for its role in lactate shuttling, in the microglia of rats on TRF HFD [23,24,25]. Our results showed that *SLC16A1* in the microglia of HFD D rats was generally higher expressed in the light phase (resting and fasting phase) than in the dark phase (active and eating phase). Further research is needed to understand exactly what this means. However, one study showed a significant correlation between *SLC16A1* and lactate concentration gradients in retinal pigment epithelial cells, suggesting that lactate transport via *SLC16A1* is regulated by the circadian clock [28]. Based on this study and our own results, we believe that *SLC16A1* in microglia may play a role in the production and/or regulation of brain Lac-Phe concentrations.

Finally, this study has some limitations. One limitation is that despite the large animal cohort, this study only included male rats. In the future, it will be necessary to perform this research in female rats and to focus on potential sex-specific phenomena. Another limitation is that Lac-Phe was measured in the hypothalamus and cortex but not in other brain regions. It may be interesting to measure Lac-Phe levels in the rest of the brain to gain more insight into the distribution of Lac-Phe across the whole brain.

## 5. Conclusions

In conclusion, we found that Lac-Phe levels in the hypothalamus and cortex changed over time within the 24 h L/D cycle. Moreover, our study indicates that brain Lac-Phe is not only induced by exercise but also by the intake of a high-calorie diet independent of exercise. Finally, we introduced microglial cells as *CNDP2+*-containing cells that could potentially play a key role in Lac-Phe production and regulation in the brain, in which the *SLC16A1* lactate transporter gene may also have an important function. Although this study helps to gain further knowledge on the presence and production of Lac-Phe in the brain, further research is needed on the potential mechanism by which Lac-Phe can reduce food intake and body weight, probably by targeting appetite-suppressing neurons.

## Data Availability

All data related to the findings of this study are available from the corresponding author upon reasonable request.
